# Next-Generation Redox Mediators: Itaconate, Nitro-Fatty Acids, Reactive Sulfur Species and Succinate as Emerging Switches in Predictive Redox Medicine

**DOI:** 10.3390/antiox15040427

**Published:** 2026-03-28

**Authors:** Luca Gammeri, Alessandro Allegra, Fabio Stagno, Sebastiano Gangemi

**Affiliations:** 1 Department of Biomedical and Dental Science and Morphofunctional Imaging, University of Messina, 98125 Messina, Italy; 2 Hematology Unit, Department of Human Pathology in Adulthood and Childhood “Gaetano Barresi”, University of Messina, Via Consolare Valeria, 98125 Messina, Italy; aallegra@unime.it (A.A.); stagnof@unime.it (F.S.); 3 Department of Clinical and Experimental Medicine, School and Operative Unit of Allergy and Clinical Immunology, University of Messina, 98125 Messina, Italy

**Keywords:** Nrf2/Keap1 pathway, redox network dysfunction, itaconate, nitro-fatty acids, reactive sulfur species, protein persulfidation, succinate signaling, ferroptosis, cuproptosis, artificial intelligence

## Abstract

Oxidative stress is no longer viewed as a random imbalance between reactive oxygen species and antioxidants, but as a failure of an integrated redox network that connects metabolism, immunity, and metal homeostasis. Classical markers such as malondialdehyde and 4-hydroxynonenal define oxidative damage, yet they cannot explain how redox adaptation occurs or fails. Over the past decade, the discovery of regulated cell-death pathways (ferroptosis, cuproptosis) and emerging metabolic signals has revealed a new generation of adaptive redox mediators—including itaconate, nitro-fatty acids, reactive sulfur species and succinate—that act as electrophilic or persulfidating regulators rather than passive by-products of oxidation. This review integrates mechanistic, biochemical and clinical evidence to define how these mediators remodel the nuclear factor erythroid 2-related factor 2/Kelch-like ECH-associated protein 1, nuclear factor kappa-light-chain-enhancer of activated B cells, and hypoxia-inducible factor 1-alpha axes, coordinate lipid–metal–sulfur cross-talk, and shape vulnerability or resistance to ferroptosis and cuproptosis. By combining deep molecular research with translational perspectives, we propose a unifying framework for predictive redox medicine based on composite biomarker panels and AI-assisted phenotyping. Understanding and quantifying these next-generation mediators will open new avenues for precision nutrition, drug development, and disease prevention—transforming oxidative-stress biology from a descriptive field into an actionable platform for human health.

## 1. Introduction

Oxidative stress was historically described as an imbalance between reactive oxygen species (ROS) generation and antioxidant defenses, mainly assessed through downstream markers such as malondialdehyde (MDA) and 4-hydroxynonenal (4-HNE) [1,2]. Oxidative stress and its markers are involved in several diseases [3,4]. Lipid peroxidation of n-6 polyunsaturated fatty acids (PUFAs) results in the generation of the bioactive aldehyde 4-HNE, a molecule capable of exerting both direct and indirect modulatory effects on intracellular signaling networks and cellular homeostasis. 4-HNE can directly compromise essential cellular processes, enzymatic activities, and organelle functionality [1]. Compounds derived from lipid peroxidation are implicated in the pathogenesis of various pathological processes, both inflammatory (e.g., inflammatory skin diseases) and non-inflammatory (e.g., neoplastic and lymphoproliferative processes) [5,6,7]. The distinctive chemical configuration of 4-HNE confers pronounced electrophilic reactivity toward macromolecules bearing thiol or amino groups, thereby facilitating the formation of Schiff bases and Michael-type adducts [2]. Covalent modification of proteins by 4-HNE frequently induces conformational alterations and functional impairment, with broad implications for physiological and pathological pathways [8]. Such adduct formation has been implicated in the modulation of insulin signaling, enzymatic regulation, mitochondrial bioenergetics, fatty acid metabolism, and tubulin polymerization [8]. Notably, 4-HNE exhibits substantially greater chemical stability compared to ROS, enabling its diffusion and interaction with molecular targets distant from its site of origin [9].

However, although oxidative stress has traditionally been framed as a simple imbalance between reactive species and antioxidant defenses, this reductionist view no longer captures the complexity of redox biology. The classical definition remains useful from a historical perspective, but it does not reflect the contemporary understanding that redox homeostasis emerges from an integrated and highly coordinated network involving metabolic pathways, electrophile–thiol interactions, metal homeostasis, and immune signaling. In this framework, ‘antioxidants’ should not be regarded as isolated molecular scavengers, but rather as components embedded within a broader redox regulatory system that includes enzymatic buffers, metabolic intermediates, lipid-derived electrophiles, and sulfur-based signaling species. Accordingly, oxidative stress is now considered a failure of this interconnected network—rather than the consequence of a binary imbalance. This perspective also provides the mechanistic basis for understanding the role of next-generation redox mediators such as itaconate, nitro-fatty acids, reactive sulfur species, and succinate, which act not as passive indicators of oxidative burden but as active regulators within the redox network.

However, the investigation of the oxidative profile remains extraordinarily complex, and findings are generally limited to a snapshot assessment of the redox state, without the possibility of fully capturing its generative dynamics. As a result, the overall effects provide only marginal value in elucidating pathological processes. The interactions between ROS and organic molecules are inherently complex, even under controlled in vitro conditions with uniform solutions. This complexity is markedly amplified in living cells, where additional factors such as membrane architecture, electrostatic forces, macromolecular binding dynamics, and the compartmentalization of enzymes, substrates, and catalytic elements introduce further layers of regulation and variability [10,11].

For these reasons, although these aldehydic products remain indispensable for evaluating lipid peroxidation, they represent endpoints of oxidative injury rather than upstream regulators of redox homeostasis, and they do not capture the real-time dynamics of oxidative stress.

Intercellular communication and responses to extracellular cues are mediated through biological processes collectively referred to as cell signaling or signal transduction [12]. Signal transduction enables the transfer of information from the cell surface to intracellular effectors, initiating functional responses. This process is typically activated by external stimuli such as hormones, growth factors, cytokines, and neurotransmitters [13]. Signals directed toward the transcriptional machinery responsible for gene expression are conveyed to the nucleus by transcription factors, which regulate RNA polymerase II activity through binding to specific DNA sequences. These signaling cascades govern diverse cellular functions, including muscle contraction, gene regulation, cell proliferation, and neuronal transmission [13].

New insights and innovative analytical approaches are therefore required to elucidate the role of oxidative stress in the transmission of intra- and intercellular signals and in the cellular response to these stimuli. In fact, although ROS are primarily associated with cellular damage, they also serve essential physiological roles in intracellular signaling and regulatory pathways [14]. Cells constitutively generate ROS, which contribute to the initiation and maintenance of signaling networks involved in growth and differentiation. Numerous cell types exhibit a transient oxidative burst, producing low ROS concentrations upon stimulation by cytokines, growth factors, or hormones [13]. This observation supports the concept that ROS act as signaling molecules within multiple levels of transduction cascades, functioning as critical secondary messengers [15].

### 1.1. Oxidative Stress in Pathological Conditions: Its Role in Cell Death

Recent studies indicate the presence of previously unrecognized signaling pathways regulated by redox mechanisms, which play a critical role in modulating physiological functions as well as in triggering and driving the progression of pathological conditions.

Oxidative stress is intricately linked to a diverse range of pathological states, including cardiovascular diseases, malignancies, neurodegenerative disorders, diabetes mellitus, ischemia/reperfusion injury, chronic inflammatory conditions, and aging [16]. These disorders can be broadly classified into two categories. The first comprises conditions in which excessive pro-oxidant activity perturbs the thiol/disulfide redox equilibrium and impairs glucose homeostasis—commonly described as ‘mitochondrial oxidative stress,’ as observed in cancer and diabetes. The second category includes diseases driven by ‘inflammatory oxidative stress,’ characterized by heightened Nicotinamide Adenine Dinucleotide (Phosphate) (NAD(P)H) oxidase activity, which promotes atherosclerosis and chronic inflammation, or by ROS generated through xanthine oxidase, a key contributor to ischemia/reperfusion injury. Aging, to a large extent, reflects the cumulative impact of free radical-induced damage, encompassing lipid peroxidation, DNA alterations, and protein oxidation [16].

### 1.2. Broader Pathophyhysiological Consequences Across Diseases

Oxidative stress exerts broad upstream effects across multiple organ systems. It contributes to endothelial dysfunction in cardiovascular disease, metabolic dysregulation in diabetes, excitotoxicity and mitochondrial impairment in neurodegeneration, and chronic inflammatory activation in autoimmune and autoinflammatory conditions [16,17]. These diverse clinical outcomes reflect the integration of ROS/RNS with complex networks involving lipid peroxidation, DNA repair signaling, inflammatory cytokine cascades, and mitochondrial respiration. Moreover, recent studies have highlighted the role of oxidative stress in genetic, metabolic, and bioenergetic signaling pathways that govern various forms of cell death, which in turn can drive the onset or progression of pathological processes [17].

### 1.3. Redox-Dependent Regulated Cell Death: Ferroptosis and Cuproptosis as Specific Outcomes

The subsequent discovery of ferroptosis, an iron-dependent form of regulated cell death driven by lipid peroxidation, and later of cuproptosis, the copper-driven mitochondrial death pathway, expanded the conceptual boundaries of oxidative pathology toward metal-dependent signalling [18,19]. It is important to clarify that ferroptosis and cuproptosis represent specific, context-dependent manifestations of redox failure, rather than the primary or exclusive expressions of oxidative stress. While they have gained substantial mechanistic relevance, they arise only under defined metabolic and metal-dependent conditions and should be interpreted within the broader network of oxidative dysregulation described above.

Ferroptosis constitutes a distinctive cell death modality characterized by iron-dependent lipid peroxidation [20]. This type of cell death has garnered increasing attention due to its pathophysiological relevance in a variety of diseases, including neurodegeneration, cancer, and ischemic organ injury [21].

Recent years have witnessed rapid progress in the mechanistic understanding of ferroptosis. Through the initial discovery of the role of the cystine-import Glutathione (GSH)–Glutathione Peroxidase 4 (GSH-GPX4) machinery in suppressing ferroptosis, the role of phospholipid hydroperoxides (PLOOHs) as the executioners of ferroptosis is now established. More recently, GPX4-independent ferroptosis surveillance pathways have been identified. Furthermore, the mechanisms of PLOOH synthesis, particularly the synthesis and activation of PUFAs, the precursor of PLOOHs, have been extensively investigated in the context of ferroptosis. Importantly, all these studies converge on cellular metabolism and have revealed an intimate relationship between ferroptosis and metabolic pathways [22].

As for cell death triggered by copper ionophores it is strongly dependent on mitochondrial respiration. Cells relying on oxidative phosphorylation are ~1000 times more sensitive to copper ionophores than glycolytic cells [23], thus indicating that cuproptosis specifically targets active mitochondrial metabolism. The morphological hallmarks of cuproptosis encompass mitochondrial shrinkage, rupture of the cell membrane, and damage to the endoplasmic reticulum and chromatin [24].

There is, nonetheless, a strong interconnection between cuproptosis and ferroptosis [25]. During cuproptosis, degradation of Fe-S clusters releases free iron ions, which elevate intracellular oxidative stress and can subsequently promote ferroptosis. Conversely, during ferroptosis, depletion of GSH and mitochondrial injury compromise cellular copper chelation and export, resulting in copper accumulation and the induction of cuproptosis. This self-reinforcing feedback loop between copper and iron metabolism has been exploited in several nanotherapeutic approaches to enhance anticancer efficacy [26,27,28].

Redox-active metals such as iron and copper are essential for critical cellular functions, including DNA synthesis, oxidative phosphorylation, ROS detoxification, and angiogenesis, while redox-inactive zinc plays structural, catalytic, and signaling roles and supports immune function. Imbalances in these metals—whether excess or deficiency—can disrupt antioxidant enzyme activity, alter sulfhydryl homeostasis, promote ROS generation (e.g., via the Fenton reaction), and trigger lipid peroxidation, DNA damage, and cell death through mechanisms like ferroptosis, cuproptosis, senescence, or inflammation. Maintaining redox homeostasis is vital for regulating signaling networks which mediate inflammatory and antioxidant responses. Metal dyshomeostasis also influences epigenetic regulation and contributes to the pathogenesis of diverse conditions, including neurodegenerative and psychiatric disorders, organ-specific diseases, and cancer [29,30,31,32].

**Figure 1:**
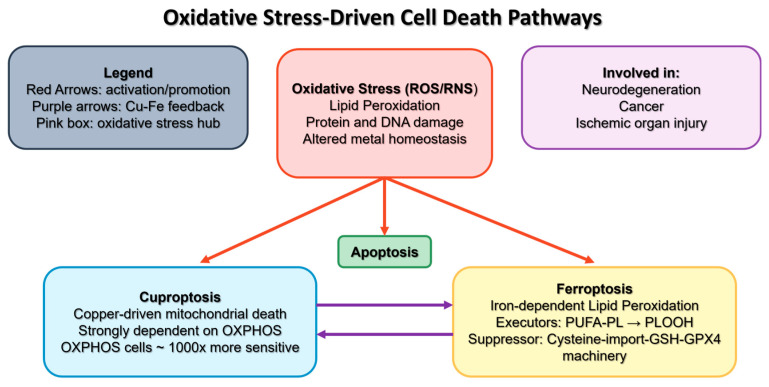
Overview of cell death pathways regulated by oxidative stress and metal dyshomeostasis. Oxidative stress (characterized by ROS/RNS, DNA/protein damage, and lipid peroxidation) acts as a central hub for activating three distinct modes of cell death: apoptosis and redox-active metal-dependent pathways, namely ferroptosis and cuproptosis. Ferroptosis is defined by iron-dependent lipid peroxidation (conversion of PUFA-PL to PLOOH) and is negatively regulated by the cystine-GSH-GPX4 axis. Cuproptosis is a copper-triggered mitochondrial death that preferentially affects cells with active oxidative metabolism (OXPHOS). The purple arrows highlight the reciprocal feedback (crosstalk) between the two processes. These signaling pathways play a critical role in the pathogenesis of neurodegenerative diseases, cancer, and ischemic organ damage.

The assessment of oxidative stress within these newly recognized dynamics of cell death is opening significant new avenues for research into disease mechanisms and therapeutic strategies. Figure 1 summarizes the main pathways involved in cell death.

These emerging forms of regulated cell deaths are characterized by distinct molecular signatures and are linked to redox imbalances, lipid peroxidation, and metal ion dyshomeostasis. Understanding how oxidative stress integrates with genetic, metabolic, and bioenergetic signaling in these contexts is essential for elucidating the pathogenesis of complex disorders, including cancer, neurodegeneration, and chronic inflammatory diseases. Furthermore, this knowledge may inform the development of targeted interventions aimed at modulating redox-sensitive pathways, thereby offering novel opportunities for precision medicine.

**Figure 2:**
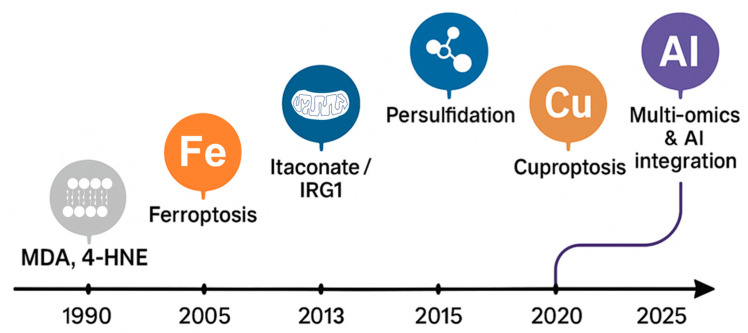
Progression from oxidative damage markers to adaptive redox mediators integrating metabolic, lipid and sulfur networks.

Metabolomic and chemical-biology studies identified new endogenous metabolites that act as adaptive electrophilic mediators, bridging metabolic and redox networks (Figure 2).

Among them, itaconate, nitro-fatty acids (NO_2_-FA), reactive sulfur species (RSS), and succinate have emerged as dynamic nodes connecting mitochondrial metabolism, immune reprogramming, and antioxidant transcriptional control [33,34,35,36].

These mediators modify cysteine-based redox sensors or metabolic enzymes, influencing master regulators such as nuclear factor erythroid 2-related factor 2/Kelch-like ECH-associated protein 1 (Nrf2/Keap1), nuclear factor kappa-light-chain-enhancer of activated B cells (NF-κB), and hypoxia-inducible factor 1-alpha axes (HIF-1α) [37,38].

This review integrates mechanistic and translational evidence on these next-generation redox switches, describing how they interlink metabolic rewiring, electrophilic signalling, and metal-dependent cell death.

By unifying biochemical foundations with clinical implications, we aim to provide a framework that advances oxidative-stress research from descriptive chemistry to predictive and personalized redox medicine.

## 2. Itaconate, Inflammation and Antioxidant Response: From Metabolic By-Product to Redox Regulator

Itaconate has rapidly emerged as a paradigmatic next-generation redox mediator, linking mitochondrial metabolism with electrophilic signaling, immunoregulation, and adaptive stress responses. Formerly regarded as a metabolic by-product of activated macrophages, itaconate is now recognized as a central node coordinating succinate dehydrogenase (SDH) inhibition, succinate accumulation, Nrf2 activation, inflammasome regulation and STING suppression.

The metabolite itaconate was first recognized in activated macrophages as a product of *immune-responsive gene 1* (*IRG1*)—now known as *aconitate decarboxylase* 1 *(ACOD1*)—which diverts citrate from the tricarboxylic-acid cycle (TCA) to form itaconate. Originally considered a metabolic by-product, itaconate was later shown to link mitochondrial metabolism to inflammation control [39].

### 2.1. Core Mechanisms and Biological Rationale

A pivotal study demonstrated that itaconate and its membrane-permeable derivative 4-octyl-itaconate (4-OI) alkylate cysteine residues on Kelch-like ECH-associated protein 1 (Keap1), releasing Nrf2 and activating antioxidant-response genes [40,41,42]. Itaconate is a critical mediator of metabolic changes as demonstrated in several studies and is produced through the conversion of aconitate by IRG1 [43,44].

Itaconate-mediated Keap1 modification facilitates the release of NF2L2/Nrf2, its translocation to the nucleus, and the regulation of genes related to antioxidant defenses as well as anti-inflammatory processes, ultimately leading to a reduction in cellular reactive oxygen species and inflammation.

Thus, itaconate is able to modify cellular activity by inhibiting succinate dehydrogenase (SDH), accumulating succinate, activating antioxidant responses, and modulating glycolytic flux, thus balancing inflammatory output and oxidative stress. This discovery reframed itaconate as an endogenous electrophile and positioned the IRG1/itaconate/Nrf2 axis as a key feedback loop in immunometabolism [45]. Indeed, targeting *ACOD1* gene expression led to reduced glycolysis but increased expression of proinflammatory associated genes, that is, *Nos2*, *IL-1β* and *IL-6* [43].

Finally, 4-OI, has been identified as a potential anti-inflammatory molecule [46] as 4-OI also affects NF-kB activation, caspase-1 activity, and Gasdermin D (GSDMD) pore formation.

Itaconate is strongly induced in inflammatory macrophages and modulates immune responses by modifying cysteine residues on target proteins. The NOD-, LRR- and pyrin domain-containing protein 3 (NLRP3) inflammasome, responsible for processing IL-1β, IL-18, and GSDMD, requires strict regulation to prevent excessive inflammation. Studies show that itaconate can modify NLRP3 and suppress its activation. Both itaconate and its derivative 4-OI inhibit NLRP3 inflammasome activity without affecting AIM2 or NLRC4. In contrast, NLRP3 activation is enhanced in IRG1−/− macrophages lacking itaconate. Mechanistically, 4-OI disrupts the NLRP3–NEK7 interaction and modifies C548 on NLRP3. Functionally, 4-OI reduces NLRP3-driven IL-1β release in Peripheral Blood Mononuclear Cells (PBMCs) from cryopyrin-associated periodic syndrome (CAPS) patients and alleviates inflammation in a urate-induced peritonitis model [47].

Recent studies have provided additional details on how itaconate coordinates metabolic rewiring and redox adaptation. Functioning as a primary metabolic regulator, this pleiotropic molecule orchestrates a spectrum of physiological and pathological processes. By covalently modifying SDH’s catalytic site, itaconate induces succinate accumulation, thereby disrupting oxidative phosphorylation and triggering a metabolic shift toward aerobic glycolysis—a phenomenon now recognized as a hallmark of inflammatory macrophage activation [48,49,50,51,52,53].

Furthermore, antioxidant activity is another key property of itaconic acid derivatives. Studies have demonstrated that itaconic acid can effectively scavenge ROS in the body, thereby reducing cell damage caused by oxidative stress [54]. By enhancing cellular antioxidant capacity, itaconic acid derivatives not only protect cells from oxidative stress damage but also improve cell function and viability. In specific experimental studies, cell models treated with itaconic acid derivatives exhibited enhanced antioxidant effects.

#### 2.1.1. Electrophilic Chemistry and Cysteine Targeting of Itaconate

Many of the aforementioned biological mechanisms of action of itaconate rely on the molecule’s electrophilic properties. As such, itaconate’s methylene group in the α position constitutes a classic Michael acceptor able to engage in addition reactions with thiols such as cysteines of proteins or glutathione [55]. For instance, the above-mentioned modification of Nrf2 has been shown to occur via proteasomal degradation of Keap1, an inhibitor of Nrf2, after alkylation of cysteine residues by itaconate [55]. A huge portion of work elucidating itaconate’s mechanisms has been done using its esterified derivatives such as dimethyl itaconate and octyl itaconate [56]. In these more electrophilic derivatives, the methylene group is more reactive, and these molecules produce biological effects distinct from those of itaconate [57].

Dimethyl itaconate (DI), 4-octyl itaconate (4-OI), and 4-ethyl itaconate (4-EI) exhibit strong electrophilic stress response, resulting in changes to the immunosuppressive phenotype. DI and 4-OI can inhibit the secretion of pro-IL-1β, IL-6, and IFN-β in an Nrf2-independent manner. In contrast, intracellular itaconate only suppressed IL-1β secretion but not pro-IL-1β levels [58].

Mechanistically, Nrf2 modification occurs through alkylation of Keap1 cysteine residues (C151, C257, C288) by the electrophilic α,β-unsaturated carboxylic acid moiety of itaconate, enhancing transcription of anti-inflammatory genes. Independently of Nrf2, itaconate and DI upregulate Activating Transcription Factor 3 (ATF3) and inhibit IκBζ, reducing IL-6 production through the ATF3–IκBζ axis, which is additionally linked to mitochondrial stress regulation [59,60,61].

Surprisingly, intracellular itaconate strongly increased the lipopolysaccharide (LPS)-induced IFN-β production. These findings indicate that itaconate derivatives do not entirely replicate the functions of endogenous itaconate due to structural and electrophilicity changes. This discrepancy also explains why itaconate and its derivatives exhibit different or even opposing effects in some experiments.

The *Stimulator of interferon genes* (*STING*) pathway is a key regulator of innate immunity, activated via cyclic GMP–AMP synthase (cGAS)-dependent or alternative mechanisms. In the canonical cGAS–STING axis, cGAMP binding induces STING conformational changes and its translocation from the endoplasmic reticulum to the Golgi. This causes recruitment of TANK-binding kinase 1 (TBK1) and IKK. These kinases phosphorylate IRF3 and IκBα, promoting nuclear translocation of IRF3 and NF-κB and subsequent expression of proinflammatory cytokines such as IFN-γ, IL-6, and TNF-α. Itaconate derivatives, notably 4-OI, attenuate *STING* signaling by activating Nrf2 and inhibiting type I IFN release; Nrf2 knockdown enhances STING expression and TBK1 phosphorylation, whereas *Keap1* silencing reduces STING levels. Furthermore, 4-OI alkylates STING cysteine residues (C65, C71, C88, C147), revealing a novel mechanism of immunomodulation by itaconate [62,63,64,65].

#### 2.1.2. Itaconate and Immunometabolic Modulation: Immunometabolic Reprogramming and Functional Consequences

Itaconate facilitates macrophage reprogramming from the proinflammatory M1 state to the reparative M2 phenotype and inhibits inflammasome activation. M1 macrophages, triggered by IFN-γ and LPS, drive early inflammatory responses, whereas M2 macrophages, induced by IL-4 and IL-13, support inflammation resolution, and tissue repair. Beyond its anti-inflammatory role in M1 cells, itaconate and its derivatives also impair Janus Kinase 1/Signal Transducer and Activator of Transcription 6 (JAK1/STAT6) signaling in M2 macrophages through covalent modification of JAK1 cysteine residues. In preclinical models of steroid-resistant asthma and lupus, 4-OI reduced M2 polarization and JAK1 activation, underscoring its potential as a therapeutic agent for M2-driven diseases [66,67,68,69,70,71].

However, determining the true biological significance of itaconate’s impact on immune system cells is of paramount importance. Immune cells reprogram metabolic pathways to cater to energy and biosynthesis demands upon activation. Most lymphocytes, including inflammatory M1 macrophages, mainly shift from oxidative phosphorylation to glycolysis, whereas regulatory T cells and M2 macrophages preferentially use the TCA cycle and have reduced glycolysis. Recent studies have revealed the “non-metabolic” signaling functions of intermediates of the mitochondrial pathway and glycolysis. The role of itaconate in immune response, including post-translational modifications of proteins and macrophages activation, have been highlighted [72].

### 2.2. Itaconate Translational Developments

Preclinical and translational studies have explored itaconate derivatives as therapeutic scaffolds in different fields such as sepsis, neurologic disease, rheumatoid arthritis. Itaconate exerts tissue-protective effects across multiple inflammatory and fibrotic conditions. Enhanced IRG1/itaconate expression in liver and lung macrophages during sepsis limits pro-inflammatory cytokine production, preserves organ function, reduces ROS, and improves GSH/GSSG balance through Nrf2 activation and NLRP3 suppression [73]. Exogenous 4-OI attenuates hepatic fibrosis, decreases oxidative stress, and limits HSC activation [74].

In murine sepsis, 4-OI improved survival, and restored glutathione balance [75]. This study examined the protective mechanisms of 4-OI in sepsis-associated acute kidney injury (AKI). In a Cecal Ligation and Puncture (CLP)-induced sepsis model, renal inflammation, oxidative stress, and ferroptosis were markedly elevated. Treatment with 4-OI or ferrostatin-1 mitigated ferroptosis, improved renal function, and exerted anti-inflammatory and antioxidant effects. Consistent in vitro findings showed that 4-OI reduced LPS-induced ferroptosis in HK-2 cells. Mechanistically, 4-OI inhibited STING pathway activation and cytokine production, partly independent of Nrf2, while also suppressing STING transcription via Nrf2 activation. These dual actions prevented STING-mediated autophagic degradation of GPX4, reduced ROS accumulation, and alleviated ferroptosis. Collectively, 4-OI emerges as a promising therapeutic candidate acting as both a STING and ferroptosis inhibitor for sepsis treatment [75].

Furthermore, notably, reduced itaconate levels correlate with increased COVID-19 severity, suggesting a protective role. These findings have prompted the development and screening of itaconate derivatives for antiviral activity, with preliminary results showing promise [76].

In autoimmune encephalomyelitis, dimethyl-itaconate reduced demyelination and cytokine storms via Nrf2 activation. Inflammatory stimuli upregulate IRG1, promoting itaconate synthesis from the TCA cycle [77]. These results support the therapeutic potential of DMI as an immunomodulatory agent for MS.

As for rheumatoid arthritis, itaconate prevents osteoclast hyperactivation by inhibiting Tet2 enzymatic activity. Moreover, exogenous administration of itaconate or its derivative 4-octyl-itaconate significantly attenuates arthritis progression and bone erosion, highlighting a potential therapeutic approach [78]. These findings establish TNF-α-driven macrophage-derived itaconate as an epigenetic regulator of osteoclast activity and position itaconate and OI as promising candidates for mitigating rheumatoid arthritis–associated bone damage.

Finally, current translational efforts focus on targeting itaconate or its derivatives to modulate cell death pathways—specifically ferroptosis and cuproptosis—as a potential strategy in oncology.

For instance, a study investigated the interplay between itaconate and cuproptosis in colorectal cancer, revealing that 4-OI promotes cuproptosis independently of other cell death pathways by inhibiting aerobic glycolysis via glyceraldehyde-3-phosphate dehydrogenase (GAPDH). This effect was attenuated by Ferredoxin 1 (*FDX1*) knockdown, and in vivo, 4-OI combined with elesclomol-Cu exhibited enhanced antitumor activity, including in oxaliplatin-resistant cells [79].

Another study revealed that Nrf2 inhibition induced by itaconate increases sensitivity to chemotherapy of colorectal cancer. Mechanistically, the effect caused by Nrf2 inhibition was closely related to the promotion of ferroptosis and pyroptosis [80].

A distinct conceptual approach is highlighted in a separate study. This experimentation showed that STAT5-driven upregulation of the IRG1/itaconate pathway in tumor-associated macrophages rewires mitochondrial metabolism and enhances Nrf2-dependent GSH synthesis, which shields tumor cells from Poly(ADP-ribose) Polymerase inhibitors (PARPi)-induced ferroptosis. Pharmacological inhibition of IRG1 restored ferroptotic sensitivity and improved antitumor immunity, highlighting the IRG1/Nrf2/GSH axis as a promising therapeutic target [81].

In conclusion, itaconate’s dual identity—metabolite and electrophile—makes it a paradigmatic redox switch bridging metabolism and antioxidant signalling. Its chemical reactivity enables reversible modulation of protein thiols, providing a flexible adaptation mechanism rather than passive radical scavenging. Therapeutic development now focuses on designing cell-permeable analogues, more suitable for crossing the blood–brain barrier, with controlled electrophilicity to fine-tune Nrf2 modification without inducing reductive stress [82].

**Figure 3:**
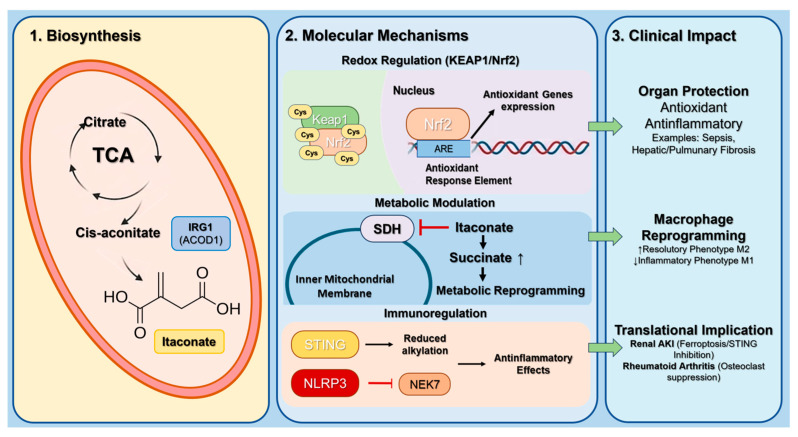
Metabolic Origin and Core Signalling Mechanisms of Itaconate. Itaconate is synthesized from cis-aconitate by the enzyme ACOD1 (IRG1) within the mitochondrial matrix. Acting as an endogenous electrophile, itaconate alkylates cysteine residues on Keap1 to trigger the Nrf2-mediated antioxidant response. Simultaneously, it inhibits succinate dehydrogenase (SDH), leading to succinate accumulation and metabolic reprogramming. Itaconate suppresses the NLRP3 inflammasome and the STING pathway, facilitating the transition of macrophages from a pro-inflammatory (M1) to a reparative (M2) phenotype. These pleiotropic effects provide multi-organ protection (liver, lung, kidney) and modulate susceptibility to metal-dependent cell death (ferroptosis and cuproptosis), offering new avenues for predictive and personalized medicine. Black arrows indicate progression or consequence. Red arrows indicate inhibition. Green arrows indicate the clinical impact of molecular mechanisms.

Future translational work will require validated assays for plasma/urinary itaconate, integration into composite redox panels, and adaptive clinical trials using redox endpoints. Figure 3 summarizes these mechanisms.

## 3. Nitro-Fatty Acids: Electrophilic Lipid Signalling

Electrophilic NO_2_-FAs are nitrated derivatives of unsaturated fatty acids formed by the reaction of nitrogen oxides with lipid double bonds. NO_2_–FAs are endogenously generated through the interaction of RNS with unsaturated fatty acids. NO_2_–FAs have been identified in human tissues, urine, plasma, and erythrocytes at concentrations ranging from picomolar to micromolar levels. These homeostatic concentrations may reflect basal oxidative stress and ongoing redox-regulated biological processes [83,84,85].

The discovery of nitro-oleic acid (NO_2_-OA) and nitro-linoleic acid (NO_2_-LA) as endogenous anti-inflammatory mediators redefined lipids as participants in redox signalling rather than passive targets of peroxidation [86].

Electrophilic lipids participate in Michael addition reactions with nucleophilic residues—primarily cysteine and histidine—within redox-sensitive proteins, resulting in reversible post-translational modifications (PTMs) that modulate enzymatic function. The olefinic nitro moiety imparts electrophilic properties to nitroalkenes at the β-carbon, thereby facilitating Michael addition to nucleophilic amino acids such as cysteine and histidine. Chromatographic and mass spectrometric analyses confirmed this reactivity in vitro and demonstrated the presence of nitroalkene-protein and GSH adducts in healthy human erythrocytes under basal conditions. Both nitro-linoleic and nitro-oleic acids formed covalent conjugates with GSH, and at physiologically relevant concentrations, nitroalkenes strongly inhibited GAPDH through modification of its catalytic cysteine (Cys-149), exhibiting IC_50_ values comparable to peroxynitrite and substantially lower than those of hydrogen peroxide. LC-MS-based proteomic profiling revealed thiol-reversible PTMs on GAPDH and GSH, increasing their hydrophobicity and promoting membrane association, which may account for previous detection difficulties [87]. Collectively, these findings suggest that electrophilic nitroalkylation represents a reversible mechanism for redox regulation of enzymatic activity, intracellular signaling, and protein trafficking in vivo.

Through these PTMs, NO_2_-FA can both activate Nrf2 and inhibit NF-κB by targeting p65, thereby coupling antioxidant and anti-inflammatory control. Nitro-fatty acids exert broad inhibitory effects on NF-κB signaling through multiple mechanisms. Nitro-oleic acid (OA-NO_2_) suppresses IκB kinase beta (IKKβ) activity, preventing IκBα degradation; alkylates RelA (p65 subunit of the NF-κB transcription factor family) at Cys38, impairing DNA binding; and promotes RelA polyubiquitination and proteasomal degradation. Proteolytic turnover of NF-κB subunits, including RelA, is essential for terminating NF-κB activation and is regulated by ubiquitin-proteasome pathways. Additionally, thiol-alkylating and S-nitrosating agents can trigger p50 degradation via Cys62 modification. Structure–activity analyses revealed that omega-5 nitroalkenes (NCE-1 and NCE-2) were the most potent inhibitors of TNFα-induced NF-κB activity, whereas increasing the distance between the omega end and the nitroalkene markedly reduced inhibitory efficacy [88].

**Figure 4:**
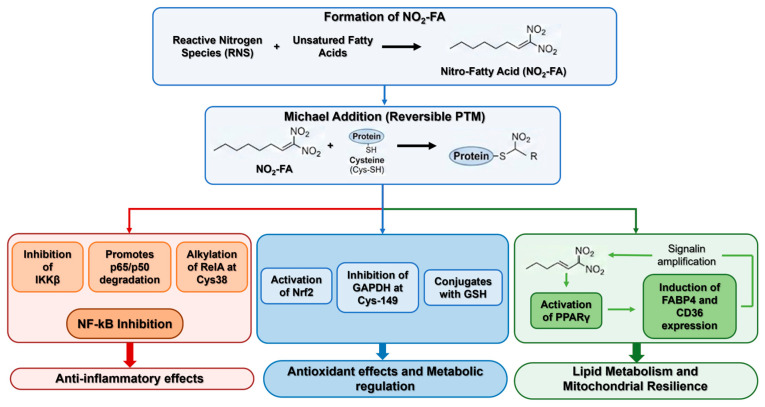
Integrative model of electrophilic NO_2_-FA signaling mechanisms. NO_2_-FAs are endogenously generated through the nitration of unsaturated fatty acids by reactive nitrogen species (RNS). These lipids possess an electrophilic β-carbon that facilitates reversible Michael addition reactions with nucleophilic thiols (cysteine residues) and histidine on redox-sensitive proteins. In the inflammatory pathway, NO_2_-FAs exert broad inhibitory effects on NF-kB signaling by suppressing IKKβ activity, directly alkylating the RelA (p65) subunit at Cys38 to impair DNA binding, and promoting subunit degradation. NO_2_-FAs modulate metabolic and redox homeostasis by inhibiting GAPDH via modification of the catalytic Cys-149 and by conjugating with glutathione (GSH). Acting as partial agonists of PPARγ, NO_2_-FAs drive the expression of FABP4 and CD36 in monocytes/macrophages. The upregulation of FABP4 facilitates a positive feedback loop that stabilizes intracellular NO_2_-FAs and amplifies downstream transcriptional activity. Collectively, these post-translational modifications couple antioxidant responses with anti-inflammatory and metabolic regulation.

They also act as partial agonists of Peroxisome Proliferator-Activated Receptor Gamma (PPARγ), enhancing lipid metabolism and mitochondrial resilience. Nitro-fatty acids function as partial agonists of PPARγ, a nuclear receptor highly expressed in adipocytes and myeloid cells. In monocytes, NO_2_-FA activated PPARγ signaling, inducing early expression of reporter genes such as *FABP4* and *CD36* during differentiation into macrophages; these effects were abolished by the PPARγ antagonist GW9662. Once fully differentiated, macrophages exhibited attenuated responses, with modest FABP4 upregulation and no CD36 induction. In vitro and in silico analyses confirmed NO_2_-FA binding to FABP4, and inhibition of FABP4-mediated fatty acid binding reduced NO_2_-FA-driven transcriptional activation of *PPARγ*-, *Keap1/Nrf2*-, and *HSF1*-regulated genes [89]. Collectively, these findings indicate that NO_2_-FA promote PPARγ activation in monocytes and enhance FABP4 expression, establishing a positive feedback loop that amplifies downstream signaling (Figure 4).

Recent mechanistic analyses confirmed that nitro-alkylation protects mitochondria from permeability transition and lipid peroxidation, reducing ferroptotic susceptibility in cardiac and hepatic cells. Results strongly suggest a positive correlation between NO_2_-OA formation and the improvement of mitochondrial function in non-alcoholic fatty liver disease (NAFLD) [90,91].

In human endothelial cultures, nanomolar NO_2_-OA up-regulated Heme Oxygenase-1 (HO-1), an inducible enzyme that degrades heme into biliverdin, free iron, and carbon monoxide. It functions as a cytoprotective and anti-inflammatory enzyme, reducing oxidative stress and modulating signaling pathways, and its expression is strongly upregulated by Nrf2 activation in response to oxidative or electrophilic stress. NO_2_-OA is also able to regulate NAD(P)H:quinone oxidoreductase 1 (NQO1), a flavoprotein enzyme that catalyzes the two-electron reduction of quinones to hydroquinones, preventing the formation of ROS through redox cycling [92].

These effects are able restoring glutathione balance and nitric-oxide bioavailability contributing to detoxification and antioxidant defense.

### Translational Evidence

Experimental models have demonstrated the relevance of these effects under pathological conditions. The potential protective effect of 10-nitrooleate against hyperoxia-induced acute lung injury (HALI) was evaluated in a C57BL/6 mouse model. Mice received intratracheal NFA administration, were exposed to hyperoxia for 48 h, and then maintained in room air for 24 h before analysis. Both intratracheal and intraperitoneal delivery of 10-nitrooleate markedly reduced inflammatory cell infiltration, alveolar-capillary leakage, and bronchoalveolar cytokine levels (IL-6, TNFα), while promoting resolution of lung inflammation. Western blotting revealed decreased NF-κB p65 expression and increased antioxidant proteins (HO-1, NQO1) in lung tissue. Furthermore, 10-nitrooleate reversed hyperoxia-induced upregulation of mitophagy markers (PINK1, p62/SQSTM1), indicating restoration of mitochondrial homeostasis. These findings suggest that 10-nitrooleate mitigates HALI/ARDS by enhancing antioxidant defenses and modulating mitophagy [92].

Furthermore, in a different animal model of metabolic disease, NO_2_-OA reduced aortic inflammation and improved vascular compliance via Nrf2 activation [93].

Using *NF-κB-luciferase* transgenic mice, a study assessed the anti-inflammatory effects of OA-NO_2_ compared to OA on LPS-induced NF-κB activation. Pre-emptive OA-NO_2_ administration significantly inhibited NF-κB activation both in vivo and in isolated macrophages. Acute intravenous delivery of OA-NO_2_ reduced leukocyte recruitment to the vascular endothelium, as visualized by intravital microscopy, and markedly decreased aortic adhesion molecule expression. Plasma concentrations in the nanomolar range were sufficient to suppress LPS-induced TLR4 cell-surface expression and NF-κB activation. In vitro, OA-NO_2_ disrupted TLR4 signaling by inhibiting IκBα phosphorylation and ubiquitination, impairing IKK phosphorylation, and preventing recruitment of TLR4 and TRAF6 to lipid raft domains. Collectively, these findings demonstrate that nitro-fatty acids exert potent anti-inflammatory effects by interfering with upstream components of the NF-κB pathway, thereby attenuating vascular inflammation through disruption of TLR4 signaling complexes [91,92,93,94].

Collectively, NO_2_-FA exemplify endogenous electrophilic therapy: molecules that redirect oxidative signalling toward adaptation rather than damage.

## 4. Reactive Sulfur Species (RSS) and Protein Persulfidation

Endogenous RSS are generated through cystathionine γ-lyase (CSE), cystathionine β-synthase (CBS), and 3-mercaptopyruvate sulfur-transferase (MPST). Reactive sulfur species—persulfides (R–SSH), polysulfides (R–S_n_–R), and sulfane sulfur—represent a parallel thiol-based redox system complementary to electrophilic carbon and nitrogen signalling. RSS, including hydrogen sulfide (H_2_S), low-molecular-weight persulfides/polysulfides, and protein persulfidation, represent a third redox axis alongside ROS and RNS. Persulfidation, was identified as a protective post-translational modification that prevents irreversible oxidation and maintains catalytic competence [95].

Emerging assays now quantify total persulfides, prodrug of persulfides and plasma RSS, proposing them as dynamic biomarkers of redox resilience [96].

Nanomolar H_2_S, generated via trans-sulfuration (CBS/CSE) and 3-MST, is oxidized by sulfide-quinone reductase to persulfides that support mitochondrial respiration while limiting superoxide formation. Dynamic persulfidation modulates cysteine-based sensors in metabolic enzymes (e.g., GAPDH), inflammatory mediators (NLRP3, p47phox), and transcriptional regulators (Keap1/Nrf2), thereby linking RSS signaling to energy homeostasis, vasodilation, innate immunity, and neuroplasticity. Dysregulated sulfur signaling—whether deficient or excessive—contributes to cardiovascular failure, sarcopenia, neurodegeneration, cancer, and post-COVID-19 syndromes [97]. Functionally, persulfidation activates Nrf2 via Keap1 cysteine modification, synergising with electrophiles such as itaconate and NO_2_-FA [97].

RSS also interact with nitric-oxide and nitro-lipid species to form S–N hybrids, contributing to vascular tone and inflammation resolution. Hydrogen sulfide, nitric oxide (NO), and carbon monoxide (CO) are now recognized as essential gasotransmitters that modulate vascular homeostasis through mechanisms involving vasodilation, angiogenesis, inflammatory regulation, and oxidative balance. Once considered toxic, these molecules are synthesized on demand by specific enzymes and their activity is governed by redox-sensitive signaling. ROS, notably superoxide and hydrogen peroxide, influence gasotransmitter biosynthesis at both transcriptional and post-translational levels and further regulate their bioavailability via oxidative modifications, including thiol persulfidation, nitrosative signaling, and carbonylation [98]. This dynamic redox control ensures a coordinated vascular response to metabolic and environmental stimuli [99].

These enzymes sustain mitochondrial bioenergetics and detoxify reactive oxygen species through H_2_S ↔ RSS interconversion.

Recent persulfidomics approaches have mapped thousands of modified proteins involved in glycolysis, antioxidant defence, and mitochondrial respiration [100].

**Figure 5:**
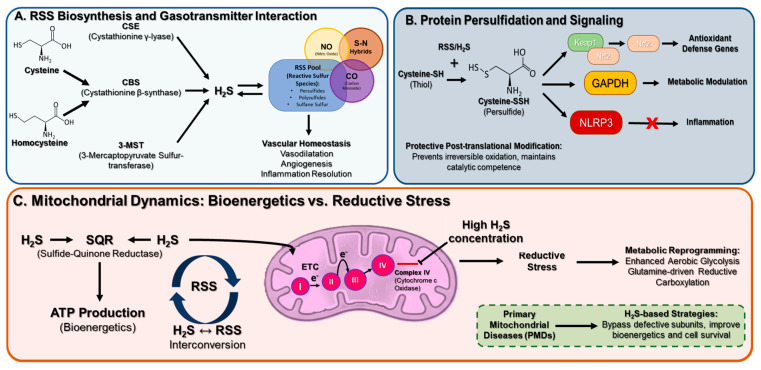
Integrative Mechanisms of Reactive Sulfur Species (RSS) in Signaling, Redox Regulation, and Mitochondrial Bioenergetics. (**A**) Endogenous H_2_S and the RSS pool are generated via the enzymatic actions of cystathionine γ-lyase (CSE), cystathionine β-synthase (CBS), and 3-mercaptopyruvate sulfurtransferase (3-MST) on cysteine and homocysteine. These sulfur species interact synergistically with nitric oxide (NO) and carbon monoxide (CO), forming S–N hybrids that collectively modulate crucial processes in vascular homeostasis, such as vasodilation, angiogenesis, and inflammation resolution. (**B**) RSS mediate signaling primarily through protein persulfidation, a protective post-translational modification converting cysteine thiols (Cys-SH) to persulfides (Cys-SSH). This modification preserves catalytic competence and prevents irreversible oxidation. Key modulatory targets include Keap1 (triggering Nrf2 nuclear translocation and antioxidant gene expression), GAPDH (regulating metabolism), and NLRP3 (inhibiting inflammatory pathways), resulting in enhanced cellular redox resilience. (**C**) H_2_S exerts a dual effect on mitochondrial function. Physiologically, it is oxidized by sulfide-quinone reductase (SQR), donating electrons to the electron transport chain (ETC) to sustain ATP production. Conversely, supraphysiological H_2_S levels inhibit Complex IV (cytochrome c oxidase), inducing reductive stress and driving metabolic reprogramming toward aerobic glycolysis and reductive carboxylation. This unique mechanism highlights H_2_S-based strategies as potential therapeutic avenues for bypassing defective subunits in Primary Mitochondrial Diseases.

Although protein persulfidation remains the central focus in elucidating H_2_S signaling mechanisms, the ability of H_2_S to induce reductive stress by targeting the electron transport chain (ETC) and reprogramming redox metabolism has only recently emerged. Unlike its nonspecific reaction with oxidized cysteines to form persulfides, inhibition of complex IV represents a distinct mode of action [101]. Studies revealing H_2_S as both an ETC substrate and inhibitor have uncovered ETC plasticity and fumarate utilization as an alternative terminal electron acceptor. Combined H_2_S oxidation and complex IV inhibition trigger mitochondrial reductive stress, propagating metabolic reprogramming characterized by enhanced aerobic glycolysis, glutamine-driven reductive carboxylation, and lipogenesis [101]. These insights mark a paradigm shift in understanding H_2_S biology, necessitating reevaluation of its physiological roles—such as cytoprotection during ischemia–reperfusion—within the framework of ETC remodeling and metabolic adaptation. Figure 5 summarizes these mechanisms.

In fact, an area of particular interest is represented by the primary mitochondrial diseases (PMDs) which represent some of the most prevalent inborn metabolic disorders, often leading to fatal outcomes within the first decade of life [102]. Their pronounced genetic and phenotypic heterogeneity poses major obstacles for targeted pharmacological interventions, as effective clinical therapies remain scarce [103]. H_2_S-based strategies have emerged as a promising avenue, given its role as a conserved mitochondrial substrate and post-translational modulator across species, with demonstrated benefits in models of age-related mitochondrial dysfunction and neurodegeneration [104,105]. H_2_S can enhance respiration downstream of commonly impaired PMD subunits, thereby improving bioenergetics, mitochondrial integrity, and cell survival [106].

Future research should explore H_2_S-driven metabolic effects in other compartments, including the endoplasmic reticulum and nucleus, and its interplay with hypoxic signaling.

### RSS and Translational Developments

In preclinical models, enhancing RSS levels—either pharmacologically (H_2_S donors) or nutraceutically (sulfur-containing amino acids)—attenuates ischemia–reperfusion injury, metabolic dysfunction, and neurodegeneration. Erucin (ERU), an isothiocyanate derived from glucoerucin in Brassicaceae plants, acts as a sustained H_2_S donor. A study examined its cardioprotective effects and underlying mechanisms, emphasizing mitochondrial Kv7.4 (mitoKv7.4) channels. ERU released H_2_S, dose-dependently protected H9c2 cells from oxidative stress, and reduced infarct size and troponin I in vivo, effects abolished by Kv7.4 blockade. In isolated mitochondria, ERU behaved as a potassium channel opener, inducing mild depolarization, limiting calcium uptake, and promoting K^+^ flux, with mitoKv7.4 persulfidation confirmed. These findings suggest ERU-mediated modulation of mitoKv7.4 contributes to cardioprotection [107].

Moreover, declines in persulfidation are linked to aging and several neurological disorders. Notably, the antioxidant regulator Nrf2 is controlled via persulfidation of Keap1. Recent evidence shows that pleozymes enhance global protein persulfidation in cells from healthy individuals and those with Friedreich’s ataxia, specifically increasing Keap1 persulfidation and promoting Nrf2 stabilization along with its downstream antioxidant targets [108].

Finally, the interplay between persulfidation and ferroptosis appears to be of particular interest. H_2_S and its derived polysulfides confer neuroprotection by mitigating oxidative stress, thereby preventing multiple forms of regulated cell death, including oxytosis and ferroptosis. Alterations in physiological levels of H_2_S and polysulfides have been implicated in the pathogenesis of various neurological and psychiatric disorders [109].

However, H_2_S enhances the susceptibility of non-small cell lung cancer (NSCLC) cells to ferroptosis, particularly under conditions of cysteine limitation. Co-treatment with H_2_S and cystine deprivation markedly potentiates ferroptosis-based therapeutic strategies. Mechanistically, H_2_S induces persulfidation of Cys195 on S-adenosylhomocysteine hydrolase (SAHH), attenuating its enzymatic function. This modification lowers homocysteine levels, thereby reducing cysteine and glutathione availability under cystine-depleted conditions, ultimately increasing NSCLC cell vulnerability to ferroptotic cell death [110]. Interesting studies have also explored the relationship between breast cancer, persulfidation, and ferroptosis [111].

Therapeutic strategies include slow-release H_2_S donors (SG1002, GYY4137), mitochondria-targeted compounds (AP39), photo- or thiol-responsive scaffolds, diet-derived polysulfides/isothiocyanates, and microbiota engineering to restore the physiological RSS window [99,112].

## 5. Succinate and Redox–Immune Signalling

Succinate, traditionally a TCA-cycle intermediate, has been recognized as an immunometabolite linking mitochondrial metabolism to inflammatory signalling. It is produced in the mitochondrial matrix and fuels ETC complex II by oxidizing to fumarate. Upon Toll-like receptor 4 (TLR4) activation, the Krebs cycle becomes truncated, reducing SDH activity and limiting succinate oxidation, which leads to its accumulation. Elevated succinate, together with increased mitochondrial ROS, stabilizes HIF-1α—a major driver of pro-inflammatory responses. Normally degraded via Prolyl Hydroxylase Domain (PHD)-mediated hydroxylation, HIF-1α persists when succinate inhibits PHDs, activating HIF-1 signaling and promoting IL-1β secretion in macrophages, potentially contributing to inflammatory disease [113,114,115,116].

Succinate Receptor 1 (SUCNR1) (GPR91), a G protein-coupled receptor for succinate, is expressed on various tissues—including kidney, spleen, and intestine—as well as on myeloid cells such as dendritic cells and macrophages. Upon succinate binding, SUCNR1′s α and βγ subunits dissociate, triggering MAPK signaling and cell-type-specific gene transcription. In immune cells, this succinate–SUCNR1 axis promotes inflammation: succinate accumulation activates SUCNR1, enhancing cytokine production in dendritic cells (DCs) and macrophages. In arthritic mouse models, extracellular succinate binding to SUCNR1 drives IL-1β secretion, while SUCNR1 deficiency reduces inflammation [117,118,119,120].

Succinate markedly enhances hMSC migration by activating a signaling cascade. It promotes pan-PKC phosphorylation, particularly PKCζ, which depends on Gαq and Gα12. PKCζ then phosphorylates p38 MAPK, leading to DRP1 phosphorylation and its translocation to the mitochondrial outer membrane, triggering mitochondrial fragmentation. This fission boosts mitochondrial function and ROS production, which activates Rho GTPases and drives F-actin formation [121].

The effects of succinate are also influenced by microenvironmental conditions. Under hypoxia or metabolic overload, succinate oxidation drives reverse electron transport (RET) at Complex I, a potent source of superoxide that aggravates tissue injury [122].

For instance, ischemia–reperfusion (IR) injury occurs when restoring blood flow to an ischemic organ paradoxically triggers oxidative damage. While reperfusion is vital for tissue survival, it causes a surge in mitochondrial ROS that drives cell death and inflammation. This ROS burst stems from succinate accumulation during ischemia and its rapid oxidation by SDH upon reperfusion, which fuels reverse electron transport at complex I and superoxide generation. SDH inhibitors like malonate can mitigate this by limiting succinate oxidation and ROS formation. To explore these mechanisms and potential therapies, an in vitro IR model was developed using isolated mitochondria exposed to anoxia with succinate, followed by reoxygenation, replicating reperfusion and enabling assessment of ROS dynamics and SDH-targeted interventions across heart, brain, and kidney mitochondria [123].

Conversely, pharmacologic SDH blockade or modulation of the SUCNR1 axis mitigates oxidative and inflammatory stress. Cardiometabolic diseases (CMD) include cardiovascular disorders driven by metabolic dysfunction, such as obesity-related cardiomyopathy, hypertensive heart disease, and diabetic cardiomyopathy. Their pathogenesis involves chronic inflammation, myocardial hypertrophy, and impaired mitochondrial energy metabolism. The succinate–GPR91 axis, beyond succinate’s role as a TCA cycle intermediate, acts as a signaling pathway influencing these processes. In metabolic conditions like obesity, hypertension, diabetes, and atherosclerosis, abnormal activation of this axis promotes inflammation, hypertrophy, and mitochondrial dysfunction, contributing to cardiovascular damage. Targeting succinate–GPR91 signaling may represent a promising therapeutic strategy for CMD [124].

Finally, clinically, plasma succinate correlates with MDA, 4-HNE, and CRP levels in metabolic-syndrome and NAFLD patients, suggesting its inclusion in composite redox–inflammatory panels [125].

Recent studies have also revealed a strong correlation between succinate and the processes of ferroptosis and cuproptosis, which may be exploited for therapeutic purposes. In fact, several investigations have evaluated the modulation of succinate levels in the treatment of cancer, renal interstitial fibrosis, and Acute Respiratory Distress Syndrome [126,127,128,129,130,131,132,133].

In conclusion, succinate dual nature—as intracellular “fuel pressure” and extracellular danger signal—makes succinate both a mechanistic driver and a measurable biomarker of redox imbalance.

**Figure 6:**
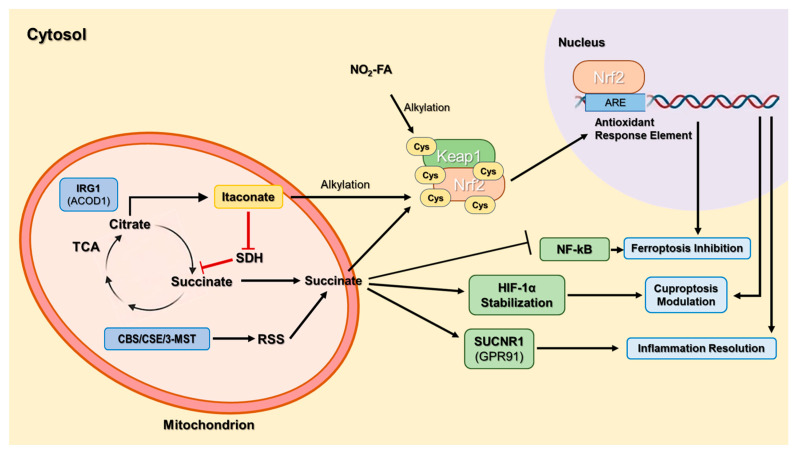
Integrated Network of Next-Generation Redox Mediators Converging on the Nrf2 Axis and Metal-Dependent Cell Death Control. The mitochondrial metabolic hub serves as a generator of key signaling molecules. Itaconate, produced by the enzyme IRG1 (ACOD1), and Reactive Sulfur Species (RSS), generated via the trans-sulfuration pathway (CBS/CSE/3-MST), are released into the cytosol. Additionally, lipid-derived Nitro-Fatty Acids (NO_2_-FA) are formed via nitration of unsaturated fatty acids. These distinct mediators converge functionally on the redox sensor Keap1. Through specific post-translational modifications of cysteine residues—alkylation (by Itaconate and NO_2_-FA) or persulfidation (by RSS)—they inactivate Keap1. This disruption prevents Nrf2 ubiquitination, facilitating its nuclear translocation. Inside the nucleus, Nrf2 binds to Antioxidant Response Elements (ARE), driving the expression of critical cytoprotective genes (e.g., GPX4, HMOX1, SLC7A11, NQO1). The resulting proteome remodels the cellular environment to inhibit ferroptosis (by limiting lipid peroxidation) and modulate cuproptosis (by regulating metal homeostasis). Concurrently, Succinate accumulates due to SDH inhibition by Itaconate, signaling independently through the SUCNR1 receptor and HIF-1α stabilization, while the electrophilic mediators exert broad anti-inflammatory effects by inhibiting the NF-κB pathway. Red arrows indicate inhibition.

Figure 6 shows the cross-talk between mitochondrial metabolism, electrophilic post-translational modifications and antioxidant gene response.

## 6. Next-Generation Redox Mediators: Translational Biomarkers and Clinical Implications

### 6.1. From Single Markers to Integrated Redox Panels

For decades, oxidative stress evaluation relied on isolated parameters such as MDA, 4-HNE, or total antioxidant capacity. However, the complexity of chronic inflammatory diseases requires multidimensional assessment combining lipid peroxidation, inflammatory, metallomic, and immunometabolic indices.

MDA is widely used to evaluate oxidative stress in inflammatory diseases, but its specificity is limited. Factors such as aging, physical activity, and diet can influence MDA levels, and it is not disease-specific, reducing its value for precise diagnosis. Composite biomarker panels—incorporating MDA/4-HNE, CRP/IL-6, Fe/Cu ratio, NADPH/NADP^+^, succinate, and next-generation mediators like itaconate, NO_2_-FA, and RSS—might allow accurate redox phenotyping and treatment monitoring [134].

These integrated signatures stratify patients into balanced, compensated, or decompensated redox states, providing a functional readout of disease activity and therapeutic response. For instance, using public glioblastoma datasets and proprietary sequencing data, a study applied computational and bioinformatics approaches to classify tumors by antioxidant capacity and assess the functional and prognostic roles of distinct transcriptional networks. Three co-expression clusters (C1, C2, C3) were identified: C1 showed strong antioxidant properties, C2 displayed an inflammatory profile and correlated with the aggressive mesenchymal subtype, while C3 had the weakest antioxidant capacity. Notably, high Gene Set Variation Analysis (GSVA) scores for the C2 signature were linked to poorer overall and progression-free survival [135].

It is therefore essential to develop increasingly sensitive techniques for quantifying novel redox activity markers that accurately reflect the dynamics of these emerging biomarkers. Plasma and serum remain the most accessible compartments for quantifying lipid peroxidation markers, dicarbonyl adducts, metallomic alterations, succinate, and emerging electrophilic mediators such as itaconate, which have been successfully measured through LC–MS/MS-based assays in both humans and animal models. Urine provides complementary information on TCA intermediates, oxidative metabolites, and mitochondrial dysfunction signatures, as shown in studies of overactive bladder and metabolic disease. Peripheral blood mononuclear cell (constitute an informative cell population for interrogating Nrf2-related antioxidant responses, inflammasome activity, and SUCNR1 expression, while tissue-specific assessments—such as liver, cardiac or neural biopsies in preclinical models—allow quantitative evaluation of redox-dependent cell-death pathways, persulfidation profiles, and nitro-alkylated proteins. Together, these matrices provide a feasible laboratory infrastructure for implementing a stepwise redox-monitoring strategy without overstating its current generalizability.

Altered itaconate metabolism has been linked to inflammatory disorders, highlighting its biomarker potential. A study established and validated a sensitive High-Performance Liquid Chromatography coupled with Tandem Mass Spectrometry (HPLC–MS/MS) assay for itaconate, its isomers, 4-octyl-itaconate, and selected TCA intermediates, achieving submicromolar detection limits. Stability studies revealed differential behavior among isomers, with anticoagulant type affecting metabolite quantification. Plasma contained citraconate but not itaconate or mesaconate, whereas LPS stimulation induced itaconate. In mice, isomer distribution was organ-specific, underscoring distinct metabolic roles. This assay enables robust quantification of itaconate isomers for biomarker and pharmacokinetic studies while providing metabolic context through TCA intermediate measurement [136].

A thorough assessment of these novel indices through the application of advanced analytical techniques is likely to enable the development of new prognostic scoring systems for a wide range of diseases. Untargeted plasma metabolomics was performed in newly diagnosed rheumatoid arthritis (RA) patients before and three months after starting conventional Disease-Modifying Antirheumatic Drugs therapy within a randomized treatment strategy trial (NCT00920478) [137]. Metabolites associated with changes in Disease Activity Score using 44 joints (DAS44) scores were identified at three months, revealing nine species strongly linked to clinical improvement, most notably itaconate, its anhydride, and an itaconyl-CoA derivative. Rising itaconate correlated with reduced DAS44 and lower C-reactive protein, implicating macrophage immunometabolism in therapeutic response. These findings provide the first evidence in humans connecting itaconate production to inflammatory resolution and support development of biomarker assays focused on the itaconate pathway [137].

Similarly, urinary metabolomic profiling in women with overactive bladder (OAB) revealed metabolic signatures of mitochondrial dysfunction, oxidative stress, and altered energy metabolism, correlating with symptom severity. Predictive modeling identified age, glycemia, and specific metabolites (malate, fumarate, α-hydroxyisobutyrate) as key determinants of OAB severity, with fumarate showing strong diagnostic performance. These findings support urinary metabolites as potential biomarkers for OAB diagnosis and severity assessment [138].

Similar considerations can be extended to other indices, such as succinate. Succinate emerges as a rapid, sex-specific biomarker of MASLD progression, rising early with steatosis and correlating with advanced disease and fibrosis. Mechanistic studies link fructose-induced succinate release to mitochondrial dysfunction and oxidative stress, reversible by Ursodeoxycholic Acid (UDCA) derivatives. The deletion of Glutaredoxin 2 (a mitochondrial enzyme involved in maintaining redox homeostasis and regulating disulfide bond formation in proteins) abolishes metabolite accumulation in males, while females remain resistant, underscoring sex-dependent metabolic regulation [139]. These findings highlight succinate’s diagnostic potential, particularly in male and pediatric MASLD.

### 6.2. Succinate and SUCNR1 as Redox–Metabolic Readouts

Quantification of plasma or urinary succinate by LC–MS/MS, coupled with SUCNR1 expression analysis in peripheral blood mononuclear cells, provides insight into mitochondrial ROS pressure and metabolic stress.

Succinate and its receptor GPR91 have been implicated in the pathogenesis of diabetic kidney disease (DKD). Hyperglycemia and hypoxia can inhibit or partially reverse succinate dehydrogenase and other TCA cycle enzymes, leading to succinate accumulation. This buildup activates GPR91, promoting renin release and early glomerular hyperfiltration, thereby triggering renin–angiotensin system activation in diabetes [140,141,142,143,144]. These findings suggest that succinate-driven metabolic alterations accelerate renal dysfunction in DKD. However, human studies remain inconsistent; for example, Feng et al. reported reduced urinary succinate in albuminuric DKD patients [145].

Elevated succinate levels correlate with high oxidative scores (MDA, 4-HNE, Fe/Cu imbalance) in NAFLD and metabolic-syndrome patients, while SUCNR1 antagonism reduces these indices in experimental models [146].

Incorporating succinate/SUCNR1 ratios into clinical phenotyping may refine prediction of therapeutic response to nutraceutical and metabolic interventions.

### 6.3. Electrophilic Lipids and Adductomics

DNA adductomics encompasses the comprehensive analysis of all DNA adducts and has advanced largely through improvements in mass spectrometry, particularly LC–MS-based tandem and multistage approaches. Currently, it serves as a powerful tool for detecting known and unknown adducts, offering insights into exposure to genotoxic agents and their molecular mechanisms. Several adducts identified through this approach have potential as indicators of adverse health outcomes. DNA adductomics has successfully uncovered exogenous carcinogens, including bacterial genotoxins and N-nitrosamines, and revealed age-dependent adduct patterns that may function as biomarkers of aging [147]. These developments underscore its utility in medicine, biology, and environmental research.

NO_2_-FAs serve as pharmacodynamic markers of electrophilic signalling and Nrf2 activation.

Targeted LC–MS/MS and protein-adductomics assays quantify free NO_2_-OA and peptide nitro-alkylation burden. Several signaling proteins involved in anti-inflammatory, antihypertensive, antihyperglycemic, and cytoprotective pathways are targets of NO_2_-FA modification, highlighting their therapeutic potential in major non-communicable diseases such as cardiovascular, renal, pulmonary, and metabolic disorders. Mass spectrometry represents a powerful approach for detecting NO_2_-FA–protein adducts; however, improved analytical strategies are needed to enhance detection sensitivity and to elucidate protein targets and signaling roles of these electrophilic lipids [148].

Clinical trials using NO_2_-OA derivatives reported concurrent rises in plasma nitro-FA and antioxidant gene expression, correlating with declines in systemic IL-6 and F_2_-isoprostanes. Beyond experimental models, understanding of NO_2_-fatty acid biology continues to evolve, particularly regarding physiologically relevant concentrations in animals and humans and their impact on cardiovascular signaling pathways. Current research focuses on cell-specific deletions of key inflammatory pathways and aims to clarify how NO_2_-FAs modulate metabolic and inflammatory processes in chronic cardiovascular disease, as well as the consequences of pathway inhibition for therapeutic efficacy [149,150,151,152].

Lipid-based therapeutics are rapidly advancing, offering new strategies for chronic conditions including cardiovascular disease. As phase II trials of NO_2_-FAs progress, pharmacokinetic and pharmacodynamic profiling, together with biomarker analysis, will guide optimal dosing. Additional efforts target slow-release formulations and selective delivery to end organs to enhance therapeutic potential.

## 7. Future Directions and Research Agenda

### 7.1. Standardization of Redox Biomarkers and Methodology

Despite major progress, the clinical use of redox biomarkers is hampered by analytical heterogeneity. Consensus initiatives now emphasize the need for harmonized protocols for sample handling, calibration, and inter-laboratory validation.

We propose unified reference procedures for MDA, 4-HNE, AOPP, and metallomic assays, providing a basis for multicenter comparability. Similar frameworks are required for itaconate, NO_2_-FA, RSS, and succinate, including reference ranges and quality-control materials.

However, a major challenge in contemporary research is the lack of methodological standardization, which limits the comparability of quantitative data across studies. From a technological standpoint, a critical component of future redox phenotyping relies on rigorous control of pre-analytical variables, which profoundly influence biomarker stability and quantitative reproducibility. Although LC–MS/MS, HPLC-based adductomics, and persulfidomics platforms enable high-sensitivity detection of itaconate, succinate, NO_2_-FA, and RSS-derived metabolites, the reliability of these measurements depends on standardized procedures for sample acquisition, processing, and storage. Freezing–thawing cycles, prolonged room-temperature exposure, anticoagulant choice, and delays in centrifugation can substantially alter redox-active metabolites and post-translational modifications, as highlighted in recent assays validating itaconate isomers and TCA intermediates, or in studies evaluating lipid nitration products and protein adducts under different storage conditions. Similar concerns apply to sulfur-based species, since persulfides and polysulfides are highly labile and require immediate alkylation or stabilization to prevent artefactual oxidation, as underscored by emerging persulfidomics methodologies. These considerations demonstrate that technological feasibility must be accompanied by stringent pre-analytical harmonization to ensure cross-study comparability.

Plasma or serum should be processed within 30–60 min, stored at −80 °C, and protected from repeated freeze–thaw cycles to preserve lipid peroxidation markers (MDA, 4-HNE), nitro-alkenes, succinate, and itaconate, all of which have shown susceptibility to degradation or isomerization under suboptimal conditions. Urine, by contrast, provides a stable matrix for TCA-cycle intermediates and oxidative metabolites, as demonstrated in metabolomic studies of inflammatory and metabolic disorders. When cellular phenotyping is required, PBMCs should be isolated promptly to avoid artefactual activation of Nrf2, inflammasome components, or SUCNR1-linked signalling pathways. For sulfur-based mediators, immediate derivatization and low-oxygen handling are essential to maintain physiological persulfidation patterns. Together, these detailed pre-analytical considerations clarify the technological foundations of our proposed approach and delineate the conditions necessary for its reliable future application.

In redox biology, several landmark papers have highlighted this issue from an analytical perspective. Beyond these considerations, other experimental practices require reassessment. Following a prolonged phase dominated by unsystematic approaches—such as indiscriminate antioxidant supplementation or dietary interventions—the field is now shifting toward rational, evidence-based designs and rigorously justified methodologies [153,154,155].

A possible solution is the integration of multi-omics and systems biology. Multi-omics integration—transcriptomics, metabolomics, proteomics, and metallomics—will delineate how nutraceuticals and metabolic mediators reprogram redox networks [156,157,158,159,160,161].

We propose a Redox phenotyping algorithm with a practical workflow for redox phenotyping integrates these mediators into a dynamic monitoring loop:

1. Baseline (T_0_): measure MDA, 4-HNE, CRP, IL-6, Fe/Cu ratio, NADPH/NADP^+^, succinate, itaconate, NO_2_-FA, RSS.
2. Classification: balanced/compensated/decompensated phenotype.
3. Intervention: apply nutraceutical or pharmacological modulation (e.g., Nrf2 activators, omega-3, CoQ10).
4. Monitoring: monthly reassessment with AI-based trend analysis.
5. Outcome: composite score reduction ≥ 30% is proposed as a benchmark for redox improvement, providing a threshold that accounts for analytical noise and ensures clinical relevance.

This adaptive model can be embedded into redox-telemetry trials for chronic inflammatory diseases, aligning laboratory metrics with patient-reported outcomes.

Although the redox phenotyping algorithm we propose is conceptually grounded in emerging multi-omics and metabolomic studies, we nevertheless believe that it cannot yet be considered a universally generalizable framework. Its applicability is most appropriate in experimental or clinical settings where oxidative stress contributes to chronic inflammatory, metabolic, or mitochondrial dysfunctions, and where multidimensional readouts have already been validated. Indeed, studies integrating oxidative biomarkers, metabolomics, and immune signatures—such as those evaluating itaconate in rheumatoid arthritis, or antioxidant transcriptional clusters in glioblastoma—demonstrate that composite redox indices can stratify disease phenotypes and correlate with therapeutic response [135,137]. Similarly, investigations of succinate accumulation in metabolic dysfunction-associated steatotic liver disease, or the mapping of sulfur-based modifications across metabolic enzymes, highlight the feasibility of using integrated panels to capture dynamic redox alterations in vivo [96,140]. Within these contexts, a structured phenotyping workflow may therefore assist in identifying compensated versus decompensated redox states, although further validation in broader populations is needed.

### 7.2. Artificial Intelligence and Predictive Modelling

Artificial Intelligence and predictive modeling are emerging as transformative tools in the study of oxidative stress, offering unprecedented capabilities for data integration and hypothesis generation. By leveraging machine learning algorithms and advanced computational frameworks, these approaches enable the identification of complex, non-linear relationships between oxidative biomarkers, genetic profiles, and clinical outcomes. Predictive models can not only stratify patient risk but also forecast disease progression and therapeutic response, thereby supporting precision medicine strategies. Integrating AI-driven analytics with experimental data holds the potential to unravel the multifactorial nature of oxidative stress and its role in pathological processes, paving the way for targeted interventions and improved prognostic accuracy [162,163].

**Figure 7:**
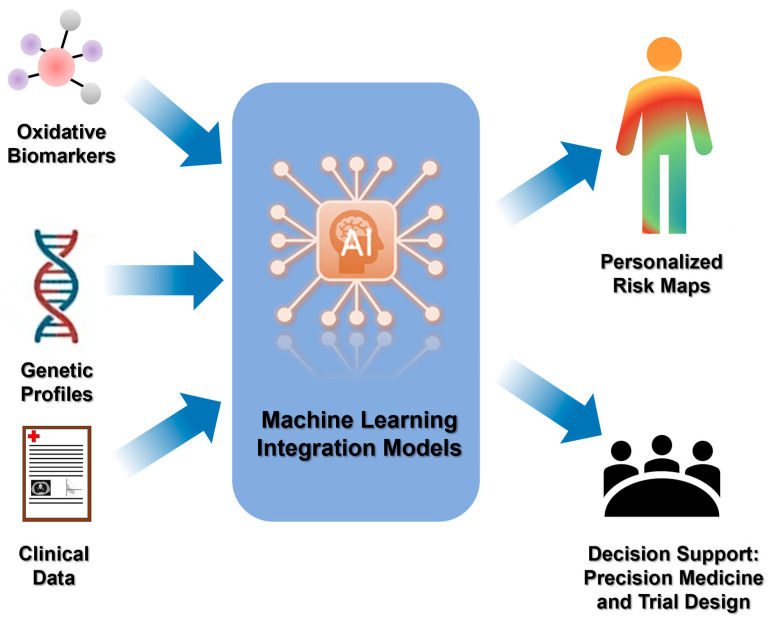
AI-Driven Redox Phenotyping for Precision Medicine. Machine learning integration models synthesize multidimensional data—including oxidative biomarkers, genetic profiles, and clinical records—to generate personalized risk maps. This AI-driven approach supports clinical decision-making and optimizes trial designs, facilitating targeted interventions and improved prognostic accuracy.

Machine-learning models trained on composite redox panels predict clinical outcomes and nutraceutical responsiveness with high accuracy [164]. A recent study identified phenethyl isothiocyanate (PEITC), a bioactive compound abundant in cruciferous vegetables such as watercress and broccoli, as a modulator of Alpha-1 antitrypsin (AAT) function [165]. PEITC enhances monomer secretion and neutrophil elastase inhibitory activity via the ER redox sensor PDIA4. Combined treatment with PEITC and an autophagy activator reduces intracellular AAT-Z polymer accumulation. Using Gaussian process–based spatial covariance mapping at atomic resolution, researchers demonstrated broad restoration of AAT variant function across the global AATD population. This approach highlights the potential of natural products like PEITC to influence ER redox and inflammatory homeostasis, offering a nutraceutical strategy for mitigating AAT deficiency-related pathology. AI approaches integrate biochemical, genetic, and lifestyle data, generating personalized risk maps and adaptive interventions. Embedding these algorithms into clinical trial design can shorten development time and improve endpoint precision. Figure 7 schematizes the ML-integrated algorithm.

### 7.3. Telemetry and Continuous Redox Monitoring

Oxidative and glycation stress are interconnected pathological mechanisms that drive the onset and progression of chronic conditions such as diabetes, kidney disease, cardiovascular disorders, and neurodegeneration. These processes induce biomolecular damage through reactive oxygen and nitrogen species and advanced glycation end products, exacerbating cellular dysfunction. Accurate biomarker monitoring is therefore critical for mechanistic insights and clinical evaluation. While conventional analytical techniques—chromatography, mass spectrometry, and immunoassays—offer high sensitivity, their widespread use is limited by cost and complexity. Emerging electrochemical and optical biosensors provide rapid, portable, and real-time detection, enabling point-of-care applications and integration into wearable devices. Advances in these technologies promise improved early diagnosis, risk stratification, and personalized medicine through simultaneous monitoring of oxidative and glycation stress [166].

Advances in biosensor technology enable real-time monitoring of redox parameters. Noninvasive monitoring of biological responses to ROS in vivo offers valuable insights into processes such as development, therapeutic efficacy, drug discovery, pathogenesis, and disease prevention. Current ROS research largely relies on in vitro models with limited translational relevance. Nanoparticles (<100 nm) provide an appealing platform for biosensing due to their small size, modular design, and favorable biocompatibility. ROS-induced intracellular signaling pathways are well characterized, and numerous genetic reporter systems using fluorescent proteins enable noninvasive detection. Building on these principles, a study developed a platform that integrates nanoparticle-linked synthetic genetic elements responsive to cellular stress signals, reporting oxidative challenges through fluorescent gene expression [167]. This technology has potential both as a research tool for quantifying oxidative stress in vivo and as a future theragnostic system for ROS-related disorders. Its fabrication uses readily available components and standard molecular biology techniques, while fluorescent protein expression can be assessed via noninvasive imaging and quantitative analysis. Overall, this nanoparticle-based reporter system provides a versatile approach for real-time monitoring of ROS responses in living organisms.

Wearable or implantable redox telemetry devices correlate biochemical signals with physiological events, opening the path to participatory oxidative-stress management [168,169].

**Figure 8:**
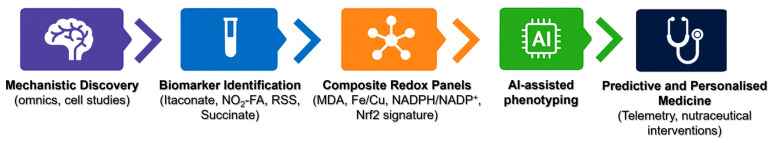
Translation pipeline linking molecular mechanism to clinical applications and continuous redox monitoring. By leveraging AI-assisted phenotyping to stratify patients based on their comprehensive redox signature, this approach enables the transition from descriptive chemistry to predictive, personalized medicine, facilitating targeted interventions and continuous monitoring via telemetry.

Pilot studies already demonstrate that continuous redox tracking predicts flare risk and therapeutic response in metabolic and inflammatory disorders (Figure 8).

## 8. Conclusions

Emerging redox mediators are increasingly recognized as actionable metabolic switches capable of reshaping inflammatory, mitochondrial and antioxidant responses. Itaconate and its derivatives activate Nrf2 via alkylation of Keap1 cysteines, suppress NF-κB-driven cytokine programs, and inhibit NLRP3 inflammasome activation, thereby protecting tissues from inflammatory and oxidative injury [40,55,57]. These molecules also modulate ferroptosis by sustaining glutathione homeostasis and preserving GPX4, and exert protective effects in models of sepsis, hepatic injury and autoimmune disorders [47,73,74,75]. Nitro-fatty acids function as endogenous electrophiles capable of inhibiting IKKβ activity, alkylating NF-κB p65, and promoting Nrf2-dependent cytoprotection, resulting in reduced vascular inflammation, improved mitochondrial function, and attenuation of lung and cardiovascular pathology [88,89,90,91,92,93,94]. Complementarily, RSS and protein persulfidation preserve catalytic cysteines, support mitochondrial bioenergetics, and regulate inflammatory sensors such as NLRP3 and p47phox, offering therapeutic opportunities in cardiovascular, neurodegenerative and metabolic diseases [95,96,97,98,99,100,101].

Beyond their intracellular biochemical actions, these mediators provide a conceptual and therapeutic framework for predictive redox medicine, as they allow targeted reprogramming of dysfunctional redox circuits rather than nonspecific ROS scavenging. Succinate, long considered a metabolic intermediate, has emerged as an immunometabolite linking mitochondrial dysfunction to inflammation via HIF-1α stabilization and SUCNR1 activation, contributing to macrophage activation, cardiometabolic injury and renal inflammation [113,114,115,116,117,118,119,120,124]. Interventions that modulate succinate levels or block SUCNR1 signaling ameliorate inflammatory and ischemia–reperfusion damage [122,123,124]. Likewise, pharmacological donors of RSS restore persulfidation networks, enhance mitochondrial resilience, and mitigate ferroptosis in neural and cardiovascular models [107,108,109,110,111]. The capacity of these mediators to influence ferroptosis and cuproptosis is now being exploited in oncology, where itaconate, NO_2_-FA, RSS and succinate-modulating strategies enhance tumor sensitivity to metal-dependent cell death and overcome therapy resistance [79,80,81,82,127,128,129,130,131,132,133]. Together, these findings position next-generation redox mediators as versatile therapeutic platforms capable of integrating metabolic rewiring, electrophilic signaling, and metal-ion homeostasis to counteract complex inflammatory, degenerative and neoplastic diseases.

The large-scale implementation of next-generation redox-based strategies will necessitate the establishment of multicenter consortia that integrate expertise across diverse domains, including redox biochemistry, nutritional sciences, bioengineering, and advanced data analytics [170]. Such collaborative frameworks should be grounded in rigorous ethical principles, prioritizing transparency, patient-centered benefit, and equitable data governance to foster trust and reproducibility. Beyond organizational aspects, these networks will play a pivotal role in harmonizing analytical methodologies and standardizing reference ranges, thereby mitigating inter-laboratory variability. This harmonization is essential for consolidating redox biomarkers as clinically actionable tools, ultimately enabling their translation from experimental paradigms to predictive and personalized medicine.

Furthermore, the integration of omics technologies—such as metabolomics, proteomics, and transcriptomics—with computational modeling will be critical to unravel the complex interplay between emerging redox mediators, including itaconate, nitro-fatty acids, reactive sulfur species, and succinate. These molecules act as dynamic metabolic switches, influencing inflammatory signaling, mitochondrial function, and cellular stress responses. Their clinical validation will require longitudinal studies and adaptive trial designs capable of capturing temporal fluctuations in redox states under physiological and pathological conditions. In parallel, the development of robust bioinformatics pipelines and machine learning algorithms will accelerate biomarker discovery and enable predictive modeling of disease trajectories. Ultimately, by fostering interdisciplinary collaboration and leveraging cutting-edge technologies, these initiatives have the potential to transform redox medicine from a conceptual framework into a cornerstone of precision healthcare.

## Data Availability

No new data were created or analyzed in this study. Data sharing is not applicable to this article.

## Abbreviations

The following abbreviations are used in this manuscript:

4-EI	4-ethyl Itaconate
4-HNE	4-hydroxynonenal
4-OI	4-octyl Itaconate
AAT	Alpha-1 Antitrypsin
AI	Artificial Intelligence
AOPP	Advanced Oxidation Protein Products
CBS	Cystathionine β-synthase
CMD	Cardiometabolic Disease
CSE	Cystathionine γ-lyase
DI	Dimethyl Itaconate
DKD	Diabetic Kidney Disease
ETC	Electron Transport Chain
GAPDH	Glyceraldehyde-3-phosphate Dehydrogenase
GSH	Glutathione
GSH-GPX4	Glutathione Peroxidase 4
MASLD	Metabolic Dysfunction-Associated Steatotic Liver Disease
MDA	Malondialdehyde
MPST	3-mercaptopyruvate sulfur-transferase
NADPH	Nicotinamide Adenine Dinucleotide Phosphate
OXPHOS	Oxidative Phosphorylation
PBMCs	Peripheral Blood Mononuclear Cell
PEITC	Phenethyl Isothiocyanate
PHD	Prolyl Hydroxylase Domain
PLOOHs	Phospholipid Hydroperoxides
PTM	Post-translational Modification
PUFA	Polyunsaturated Fatty Acid
RET	Reverse Electron Transport
RNS	Reactive Nitrogen Species
ROS	Reactive Oxygen Species
RSS	Reactive Sulfur Species
SDH	Succinate Dehydrogenase
SQR	Sulfide-Quinone Reductase
SUCNR1	Succinate Receptor 1
TCA	Tricarboxylic-acid Cycle
TLR4	Toll-like Receptor 4
UDCA	Ursodeoxycholic Acid
